# GNSS-R with Low-Cost Receivers for Retrieval of Antenna Height from Snow Surfaces Using Single-Frequency Observations

**DOI:** 10.3390/s19245536

**Published:** 2019-12-14

**Authors:** Simone Rover, Alfonso Vitti

**Affiliations:** DICAM, University of Trento, 38100 Trento, Italy; simone.rover@unitn.it

**Keywords:** GNSS reflectometry, snowpack characterization, low-cost GNSS receivers, GNSS single-frequency signals

## Abstract

Snowpack is an important fresh water storage; the retrieval of snow water equivalents from satellite data permits to estimate potentially available water amounts which is an essential parameter in water management plans running in several application fields (e.g., basic needs, hydroelectric, agriculture, hazard and risk monitoring, climate change studies). The possibility to assess snowpack height from Global Navigation Satellite Systems (GNSS) observations by means of the GNSS reflectometry technique (GNSS-R) has been shown by several studies. However, in general, studies are being conducted using observations collected by continuously operating reference stations (CORS) built for geodetic purposes and equipped with geodetic-grade instruments. Moreover, CORS are located on sites selected according to criteria different from those more suitable for snowpack studies. In this work, beside an overview of key elements of GNSS reflectometry, single-frequency GNSS observations collected by u-blox M8T GNSS receivers and patch antennas from u-blox and Tallysman have been considered for the determination of antenna height from the snowpack surface on a selected test site. Results demonstrate the feasibility of GNSS-R even with non-geodetic-grade instruments, opening the way towards diffuse GNSS-R targeted applications.

## 1. Introduction

Initially developed for positioning, navigation and timing purposes, Global Navigation Satellite Systems (GNSS) nowadays are being widely used in many and different application fields, e.g., in geodesy, geodynamics, tropospheric and ionospheric sensing and monitoring, geomatics and surveying, space applications and so on. On one hand, precise point positioning (PPP) and differential positioning (DP) models are adopted on a standard base in high precision applications, on the other, GNSS instruments operate as sensors for observation and further understanding of many natural phenomena. For an in-depth discussion about GNSS fundamentals, models and applications see [1] and references therein and also [2] for GNSS in environmental sensing.

One of the first applications of GNSS in a non-conventional field was presented by Martin-Neira in 1993 [3]. For the study of oceanic surface Martin-Neira presented a methodology focused on Global Positioning System (GPS) signals reflected by the water surface rather than on direct signals emitted by satellites only. Disturbances induced by signal reflections were related to the distance between the GPS antenna and the reflective surface.

In the following years, many further studies focused on the possibilities offered by reflectometry, not only in the oceanographic field [4,5,6] but also for other purposes such as determination of soil moisture [7,8], ice and snow monitoring [9,10,11,12,13,14,15,16,17,18] and as a remote sensing tool for agriculture [19]. In the early studies specific instruments setup were realized, in particular involving antennas with opposite polarization: one mounted in a standard “face-up” way for the detection of direct signals and one mounted “face-down” for the detection of reflected signals. Nowadays, in what is knows as GNSS Reflectometry (GNSS-R), measurements from a single antenna with standard pointing are being used to collect observations of the interference between reflected and direct and signals [1,20,21,22].

In standard GNSS-R applications, geodetic-grade GNSS instruments are used, in particular, from existing Continuously Operating Reference Stations (CORS). This kind of station is equipped with high quality antennas and receivers with specific hardware (antenna radiation patterns and physical shielding), firmware solutions and technologies specifically designed to suppress or mitigate as much as possible multi-path effects on the GNSS measurements [23,24,25,26,27,28,29]. The implementation of solutions of that kind essentially implies a degradation of the quantity which one try to exploit more in GNSS-R. Low-cost GNSS instruments are equipped with less powerful multi-path mitigation technologies. On the other hand, electronic components of low-cost instruments are of lower quality with respect to those of geodetic-grade instruments.

On the basis of seminal works leaded by Kristine Larson [10,11,14] in this work u-blox receivers with u-blox and Tallysman patch antennas were tested, along with Leica receivers and antennas, to evaluate the performance of GNSS-R in retrieving antenna heights above snowpack surface from single-frequency observations. GNSS acquisition campaigns were performed in *ad-hoc* selected sites and for relatively short time acquisition sessions. For short acquisition sessions snow density and snow complex permittivity can be considered constant. In this work, a few centimeters away form antennas installed in conventional position (face-up), other antennas were mounted face-down to empirically investigate the possibility to exploit some portions of antenna radiation patterns once reversed.

Computations were performed using originally developed code in Matlab language along with existing Matlab functions. Standard GNSS software, such as teqc [30] and gfzrnx [31], were used to ease as much as possible the processing of observations files from many receivers and observations files in both RINEX 2.x and 3.x format.

In the next section an overview of GNSS-R principles is provided. Then data collection and processing are presented. Results and Discussions sections close the paper.

## 2. GNSS-R in Short

**Figure 1:**
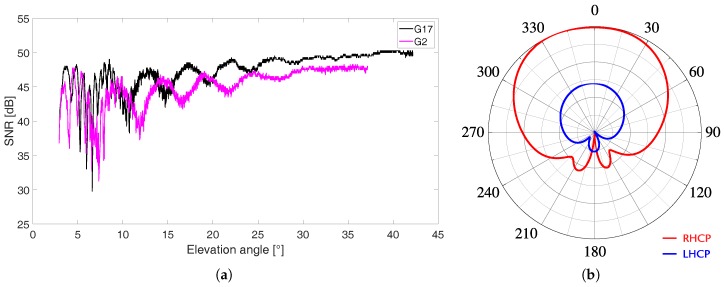
Schematic representation of (**a**) radiation patterns of a GNSS antenna and (**b**) samples of SNR observations on L1 band acquired using geodetic-grade instruments.

GNSS signals are Right Hand Circularly Polarized (RHCP) after a reflection polarization changes so that reflected signals are Left Hand Circularly Polarized (LHCP) [1,21]. The interference between direct and reflected signals produce a characteristic disturbance visible on Signal-to-Noise Ratio (SNR) data, usually used as a proxy of signal quality, see Figure 1a. GNSS user antennas present a dual polarization, RHCP for direct signals and LHCP for reflected signals, and specific radiation patterns for each polarization. Radiation patterns are optimized to facilitate omnidirectional acquisition of RHCP signals (high gain) and to reduce antenna sensitivity to LHCP signals (low gain). In Figure 1b RHCP and LHCP radiation patterns for L1 frequency are sketched.

In general, for elevation angles greater that around 10–15 degrees, the RHCP gain is larger than the LHCP gain. However, for low angles the differences become smaller and for very small or negative angles the radiation patterns are designed to be as low as possible. Beside the interference impact, SNR values depend on satellite elevation angle. The SNR signal can be seen as the result of the sum of a signal with high amplitude and low frequency (direct component, $SNR_{D}$) and a signal with low amplitude and high frequency (reflected component, $SNR_{R}$). The high frequency signal presents a lower amplitude due to design features of radiation patterns and appears more clearly at low elevation angles. For high elevation angles $SNR_{R}$, amplitude is lower than observations noise while for low satellite elevations the multipath effect is clearly visible.

To remove the $SNR_{D}$ component and isolate the signal generated by surface reflections one should model antenna radiation patterns which are rarely released to users. As an alternative, the amplitude of the direct component can be removed after a low order polynomial fitting exposing the SNR component due to multipath ($SNR_{R}$).

**Figure 2:**
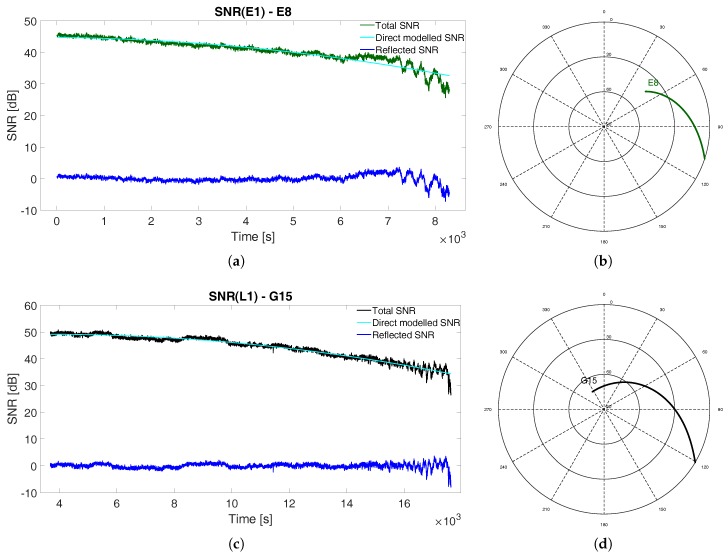

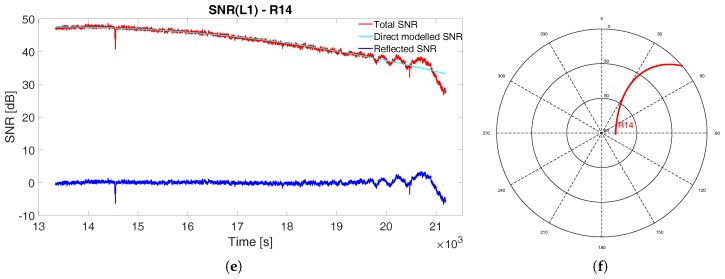
SNR observations with modeled direct component and residual reflected component for descending tracks of (**a**) Galileo E8 (signal E1), (**c**) GPS G15 (signal L1) and (**e**) GLONASS R14 (signal L1) satellites with relative skyplots for (**b**) Galileo E8, (**d**) GPS 15 and (**f**) GLONASS R14 satellites.

In Figure 2 typical SNR data for Galileo, GPS and GLONASS signals for descending tracks are reported along with samples of direct and residual reflected SNR components decoupling.

The relationship between SNR values, amplitude and phase of direct and reflected signals can be easily derived in the case of horizontal reflector exploiting a polar representation of amplitude-phase quantities. In this case the following equality holds:

(1) $$SNR^{2}=A_{D}^{2}+A_{R}^{2}+2A_{D}A_{R}\cos\Delta \phi$$

where $A_{D}$ and $A_{R}$ are the amplitudes of direct and reflected signals, $\phi_{D}$ and $\phi_{R}$ are the phases of direct and reflected signals, and the quantity $\Delta \Phi =\phi_{R}-\phi_{D}$ is the multipath relative phase. According to the amplitude difference between direct and reflected signals, see Figure 2, when $A_{D}\gg A_{R}$ Equation (1) can be simplified as:

(2) $$SNR^{2}=A_{D}^{2}+2A_{D}A_{R}\cos\Delta \phi .$$

In the case of the horizontal reflector, the additional path covered by a reflected signal relative to the path covered by a correspondent direct signal can be computed knowing the antenna height above the reflective surface and the elevation angle of the direct signal. From the geometry of a satellite–reflector–antenna configuration the phase difference Δϕ can be written as:

(3) $$\Delta \phi =\frac{2\pi }{\lambda }\;\delta$$

where

(4) δ=2hsin(θ)

is the additional path, *h* is the antenna height, θ is the satellite elevation angle and λ is the signal wavelength.

Substituting Equation (3) in (2) one get the observation equation relating SNR values to the antenna height which is the main unknown in GNSS-R applications.

Since terms of Equation (3) are time dependent the time derivative of Δϕ reads:

(5) $$\frac{d\Delta \phi }{dt}=\frac{4\pi }{\lambda }\;\left[\frac{dh}{dt}\;\sin\left(\theta \right)+h\;\cos\left(\theta \right)\;\frac{d\theta }{dt}\right].$$

Satellite elevation angle, and its time derivative, can be computed from orbital information in the satellites navigation message.

According to Equations (2) and (5) reflections from objects located near the antenna generate small SNR fluctuations whereas reflections from objects located farther from the antenna generate bigger SNR fluctuations.

By a change of variable from *t* to s=sin(θ(t)) it is possible to rewrite Equation (5) to express frequency of SNR fluctuations as a function of satellite elevation angle:

(6) $$\frac{d\phi }{ds}=\frac{4\pi }{\lambda }\left[\frac{dh}{ds}\;\tan\left(\theta \right){\left(\frac{d\theta }{ds}\right)}^{-1}+h\right].$$

When the time derivative of antenna height can be neglected with respect to other therms, the frequency of SNR fluctuations due to multipath is proportional to *h*:

(7) $$\frac{d\phi }{ds}=\frac{4\pi }{\lambda }\;h.$$

The quantity $f_{M}=2h/\lambda$ can be interpreted as the multipath frequency relative to a complete semi-arc of a given satellite trajectory, i.e., for θ∈[0,π/2]. For a generic portion of a satellite arc, with $\theta \in [\theta_{min},\theta_{min}]$ one get:

(8) $$h=\frac{\lambda f_{M}}{2\left[\sin\left(\theta_{max}\right)-\sin\left(\theta_{min}\right)\right]}$$

which expresses the antenna height above the reflective surface as a function of the multipath frequency.

In practice, the multipath frequency can be determined by means of the Lomb Scargle Periodogram (LSP) of SNR data [32,33,34,35,36]. SNR data are evenly sampled in time, but the corresponding sin(θ) values are unevenly distributed. As a matter of fact the LSP is largely adopted for detecting and characterizing periodicity in unevenly spaced data. According to LSP method the signal power spectral density is:

(9) $$P\left(f\right)=\frac{1}{2\sigma^{2}}\left\{\frac{{\left[\sum_{j}\left(h_{j}-\bar{h}\right)\cos\left(2\pi f\right)\left(t_{j}-\tau \right)\right]}^{2}}{\sum_{j}\cos^{2}\left(2\pi f\right)\left(t_{j}-\tau \right)}+\frac{{\left[\sum_{j}\left(h_{j}-\bar{h}\right)\sin\left(2\pi f\right)\left(t_{j}-\tau \right)\right]}^{2}}{\sum_{j}\sin^{2}\left(2\pi f\right)\left(t_{j}-\tau \right)}\right\}$$

where $\bar{h}$ and $\sigma^{2}$ are the mean and the variance of the observed sequence $h_{j}$, $t_{j}$ are the sample epochs, *f* denotes the frequency under consideration, and τ is a temporal offset computed from the following expression:

(10) $$\tan\left(4\pi f\tau \right)=\frac{\sum_{j}\sin\left(4\pi ft_{j}\right)}{\sum_{j}\cos\left(4\pi ft_{j}\right)}.$$

In practical application of the LSP-based spectral analysis, a set of frequencies $f_{d}$ must be constructed according to specific criteria and algorithms [36,37]. Once the frequency set has been entirely investigated, the LSP can be built and analyzed: highest value of P(f) denotes the dominant frequency which, in GNSS-R applications, corresponds to $f_{M}$.

The reflection of an electromagnetic signal is a complex phenomenon that depends on several aspects, among which signal wavelength, dielectric properties of the reflective medium, geometric features of the reflective surface and signal incident angle are of primary importance. Here, surface roughness is relative to the signal wavelength and the signal incidence angle according to the Rayleigh roughness criterion. If the incident wave is reflected mainly in one direction the reflection is referred to as a specular reflection. When the reflected wave is scattered, the phenomenon is called diffuse reflection.

GNSS signals are transmitted with an aperture angle of about a ten of degrees so that a region of the reflective surface, rather than a single point, is illuminated by the signal that reaches the antenna after being reflected. The reflective regions are known as Fresnel zones [38] which are the ellipses generated from the intersection of the reflecting surface and a family of ellipsoids having the satellite position and the mirrored antenna point as foci. The first Fresnel zone is the one that contributes most to the reflection of the incident signal.

Geometric parameters of Fresnel ellipses are completely determined from the geometry of the satellite-reflector-antenna configuration. For the first Fresnel ellipse, in the case of horizontal reflector, the ellipse center *C*, the semi-major *a* and semi-minor *b* axes are given by:

(11) $$\begin{array}{c} C=\frac{h}{\tan(\theta )}\left(1+\frac{\lambda }{2\sin(\theta )}\right)\\ a=\frac{1}{sin(\theta )}\sqrt{\frac{\lambda h}{\sin(\theta )}+{\left(\frac{\lambda }{2\sin(\theta )}\right)}^{2}}\\ b=a\sin(\theta ). \end{array}$$

## 3. Data Collection and Processing

In this work data were collected over a set of three GNSS survey campaigns. Due to hardware and power supply limitations, relying on battery units operating at low temperatures, GNSS campaigns were relatively short. Short sessions do not permit to observe real changes of the snowpack depth. To overcome this limitation, antennas were first mounted at a known height above the snowpack surface. Antenna heights were then manually changed to other know values.

**Figure 3:**
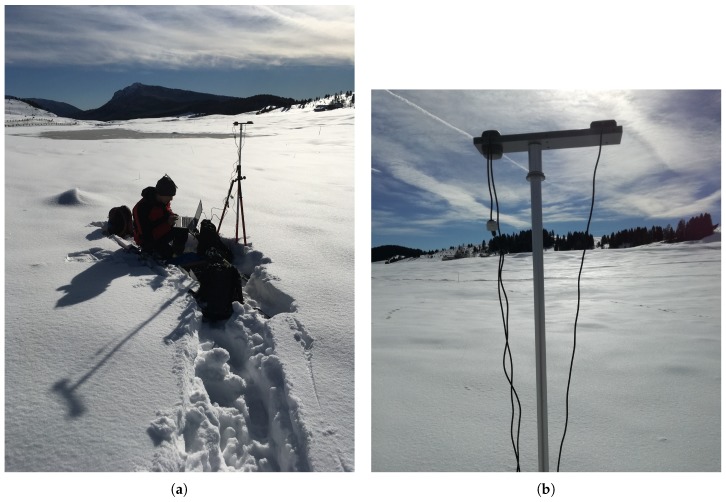
Pictures of (**a**) field setup with (**b**) low-cost patch antennas.

The first campaign took place on March 2018 with geodetic-grade GNSS antennas and receivers; antennas heights were changed of 13 cm. The second campaign took place on February 2019 with low-cost GNSS antennas and receivers; antennas heights were changed of 15 cm. Figure 3 reports a close-up view of the experimental setup during GNSS campaigns with low-cost instruments.

All the GNSS campaigns were conducted on the Lavarone plateau in the Province of Trento, Italy, at about 1400 m above see level. GNSS data were collected on a site with a wide smooth horizontal snowpack surface, in particular in the East thru South-West directions. However, in other directions the snowpack surface was much more complex and a paved road ran at the North of the antennas site. A suitable GNSS data selection was performed in order to consider only those signal reflections occurred on the smooth horizontal regions of the snowpack surface.

To empirically investigate the possibility to exploit antenna radiation patterns once reversed, for both classes of GNSS instruments, a reference antenna was installed in a conventional position (face-up) and a secondary antenna was mounted right below the reference antenna in a face-down setup.

Geodetic-grade instruments were Leica GX1230GG receiver with AX1202GG antenna and a Leica SmartAntenna ATX1230GG. Leica firmware store SNR with a 0.25 dB resolution.

u-blox NEO-M8T receivers were used along with u-blox ANN-MS and Tallysman TW4721 patch antennas. Factory firmware (version 3.1) of u-blox NEO-M8T receivers stores only integer values of SNR. To overcome this limitation receiver firmware were downgraded (Firmware 2.1 permits to track GPS signals only). from version 3.1 to version 2.1 in order to have SNR values stored with a 0.25 dB resolution. All the receivers operated at 1 Hz.

Binary files stored by receivers were converted in the Receiver INdependent EXchange (RINEX) format using UNAVCO *teqc* software [30]. When necessary, conversions between different versions of RINEX format were performed using GFZ *gfzrnx* software [31].

In this work, GNSS data processing (from SNR to satellite elevation angles), computation of Fresnel ellipses, 1D and 2D plotting, LSP implementation and analysis were performed with original code written in Matlab language.

**Figure 4:**
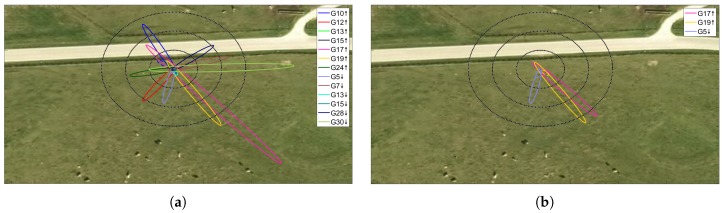
Example set of first Fresnel ellipses (**a**) before and (**b**) after data selection according to azimuth, elevation and acquisition length criteria. The black circles have radii of 5, 10 and 15 m.

Time series of SNR data and satellite elevation angles were divided in order to obtain data set for ascending and descending satellite arcs. In order to avoid processing of potentially disturbed signals, to limit the extension of reflective regions and to disregard low informative SNR data associated to high satellite elevation angles only azimuth angles in the range 90–200 degrees and elevation angles in the range 5–25 degrees where selected for further processing. To ensure the presence of typical SNR oscillations caused by multipath, only SNR time series longer that 900 s and presenting at least three SNR multipath cycles were selected. In Figure 4 sample sets of first Fresnel ellipses are reported before and after data selection according to azimuth, elevation and acquisition time length criteria.

Before performing the spectral analysis (LPS), selected SNR data were smoothed by means of a Gaussian-weighted moving average in order to reduce impact of instrumental noise in LSPs. Then a low-order polynomial fitting of the smoothed SNR data was performed in order to model the direct component $SNR_{D}$ and expose the reflected component $SNR_{R}$.

The dominant frequency $f_{M}$ can be computed by means of a numerical implementation of Equations (9) and (10) and once a suitable family of frequencies $f_{d}$ is at hand. To built the $f_{d}$ set one must choose the minimum frequency and the maximum frequency to be investigated and the frequency resolution for spanning the selected range of frequencies. These quantities can be selected according to analytical criteria developed in the spectral analysis framework for uneven samples. In practice the frequency resolution in related to the spatial resolution with which the antenna height can be resolved from the SNR analysis. The frequency resolution depends on the time length *T* of the SNR series under investigation and on a specific parameter named oversampling factor $o_{f}$. The role of this parameter is to permit to resolve with good resolution a single cycle of the frequency 1/T. The minimum frequency is, in general, set equal to the selected frequency resolution. The choice of the maximum resolution is particularly complex since it is related to the extension of the Nyquist frequency to finite uneven samples and depends on a specific parameter named max frequency factor $m_{f}$. In practice, the role of this parameter is to set the upper bound of detectable antenna height [35,36].

In our case, the value of $o_{f}$ was chosen to resolve antenna height at the centimeter level. The value of $m_{f}$ was chosen by taking in to account that antenna height above the snowpack surface cannot be greater than 2 m.

Since LSPs may manifest many peaks, in order to discard poor LSPs, only periodograms with dominant peak amplitude greater than 2.5 times the amplitude of the second peak were selected for the estimation of antenna height my means of Equation (8).

**Figure 5:**
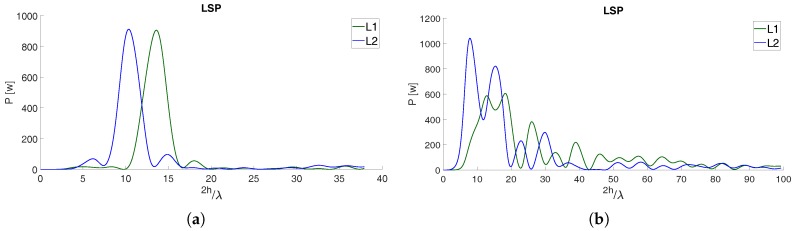
Spectral analysis applied to SNR series recorded by the same instrument but relative to different satellites and different GNSS L-band frequencies. In (**a**) is represented the result of ideal reflections whilst in (**b**) the presence of multiple peaks suggest the presence of several reflecting surfaces.

Figure 5 shows LSPs for SNR data recorded by the same instrument but relative to different satellites and different GNSS L-band frequencies. LSPs on the left present clear dominant peaks while LSPs on the right manifest many peaks. LSPs similar to those of Figure 5b were discarded from further analysis.

## 4. Results

### 4.1. Geodetic-Grade Receivers and Antennas

During the first GNSS campaign (March 2018), after a first session of about 120 min, antenna heights were shifted downwards of 13 cm from starting value $h_{0}$ = 1.29 m to new value $h_{1}$ = 1.16 m (face-up configuration) and a second session of about 120 min was carried out.

For standard mounted antennas, differences between reference and GNSS-R based antenna heights were, in general, very small and consistent as shown in Table 1. In the experiment, 12 SNR series were obtained, five for the antennas in the starting position and seven for the antennas in the lower position. Results are of good quality, in particular considering the short acquisition periods (i.e., the low number of LSPs at hand).

Worse results were obtained for the antenna mounted in face-down configuration. Only three valid SNR series were available, all for the antenna in the lower position. Differences are in the order of a ten of centimeters with a maximum value of 35 cm.

**Figure 6:**
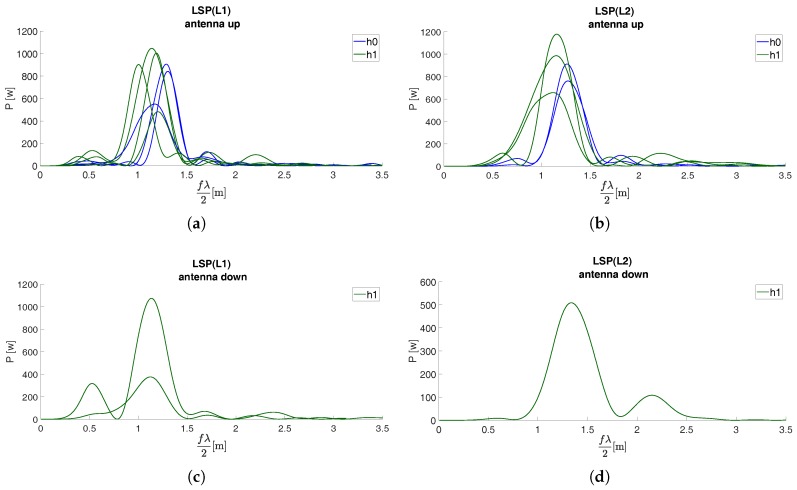
Geodetic-grade instruments: LSPs of SNR for (**a**) L1 band and face-up antenna, (**b**) L2 band and face-up antenna, (**c**) L1 band and face-down antenna, (**d**) L2 band and face-down antenna [GNSS session March 2018].

The set of LSPs for the first acquisition campaign are reported in Figure 6 for both the antenna configurations and GNSS L-bands.

### 4.2. Low-Cost Receivers and Antennas

**Figure 7:**
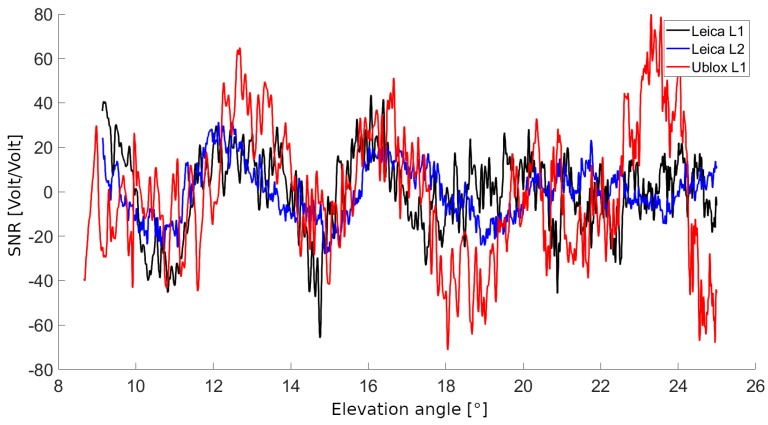
Example of $SNR_{R}$ data acquired by Leica and u-blox instrumentation tracking the same satellite.

Low-cost GNSS instruments differ from geodetic-grade solutions essentially at firmware, hardware and electronic levels. At the very beginning of this work, a test session was conducted to collect SNR data with GNSS instruments of both classes operating simultaneously in order to evaluate the quality of SNR series collected by low-cost instruments with respect to reference SNR series collected for the same satellite set by geodetic-grade instruments. Example $SNR_{R}$ series shown in Figure 7 manifest clear similarity in signal amplitude and multipath patterns supporting further investigation about the use of low-cost receivers and antennas for GNSS-R application for snowpack characterization.

During the second GNSS campaign (2019/02), after a first session of about 90 min, antenna heights were shifted downward of 15 cm from starting value $h_{0}$ = 1.71 m to new value $h_{1}$ = 1.56 m (face-up configuration) and a second session of about 90 min was carried out.

In the campaign, observations from a total of 3 antennas were acquired: 2 Tallysman TW4721 antennas, one in a face-up and one in a face-down setup, and 1 u-blox ANN-MS antenna in face-up configuration.

In the experiment, as shown in Table 2, 17 SNR series were obtained in face-up configuration. Antenna heights estimated from LSPs were characterized by a greater dispersion with respect to those obtained using geodetic-grade instruments. However average differences obtained from different SNR series differ of about 10 and 5 cm from the two antenna height reference values. Results for u-blox receivers and patch antennas are still of good quality, in particular considering the short acquisition periods.

**Figure 8:**
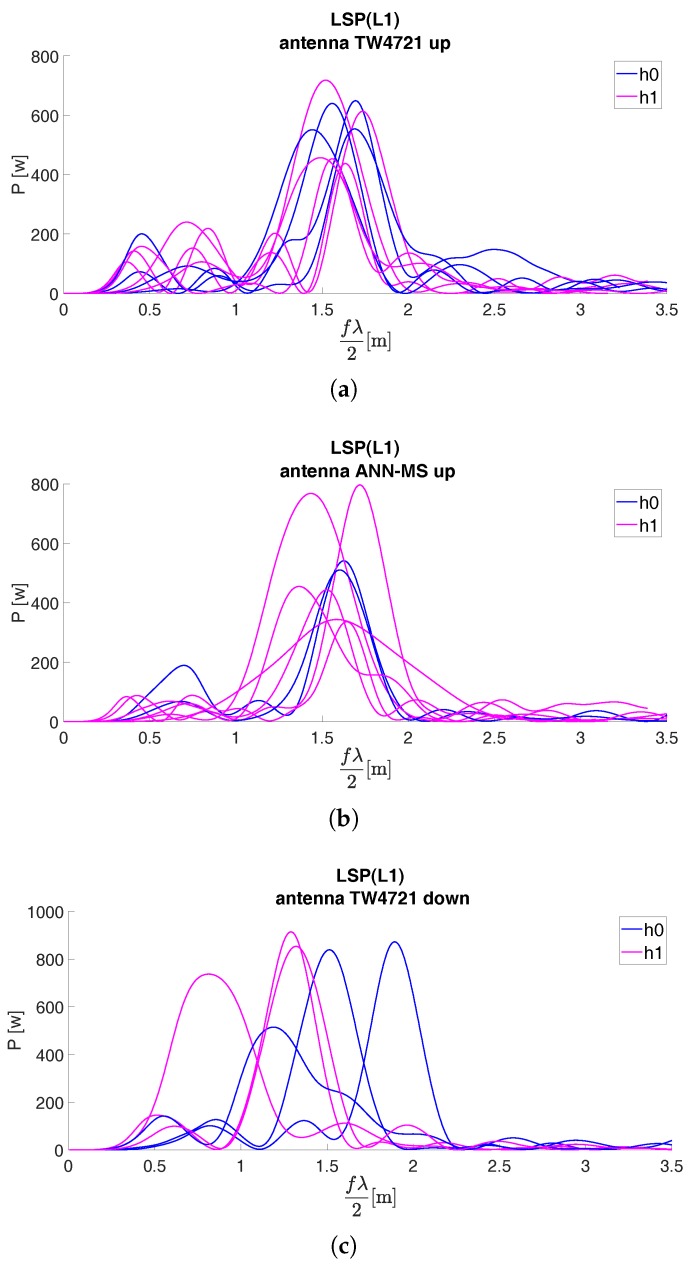
Low-cost instruments: LSPs of SNR for (**a**) L1 band and face-up TW4721 antenna, for (**b**) L1 band and face-up ANN-MS antenna, for (**c**) L1 band and face-down TW4721 antenna [GNSS session February 2019].

The set of LSPs for the second acquisition campaign are reported in Figure 8 for both the antenna configurations and GNSS L1 band.

Even for low-cost instruments results obtained with the face-down configuration are of very low quality, see Table 3.

## 5. Discussion and Perspectives

Results based on SNR observations acquired by geodetic-grade instruments showed GNSS-R effectiveness in retrieving accurate values of antenna heights from the snowpack surface. Small variations of antenna height, in the order of a ten of centimeters, can be clearly detected.

In this work, it has been shown that even GNSS-R based on SNR observations acquired by low-cost receivers and antennas can provide very good results. In this case, accuracy of antenna heights from the snowpack surface can reach quality levels of a few centimeters even if results present lower consistency with respect to values obtained from the analysis of observations from geodetic-grade instruments.

Results from antenna mounted in a face-down position demonstrate the inefficacy of this configuration, very few SNR series were collected and very inaccurate antenna height values were obtained from the LSP spectral analysis os SNR data. An alternative configuration is under investigation where four antennas are mounted with the patches placed on vertical planes plus one antenna mounted in the standard face-up position.

In conclusion, GNSS-R technique with low-cost receivers and antennas can be effectively adopted to retrieve antenna height above a snowpack surface with a satisfactory degree of accuracy and good precision (standard deviation of about 5 cm for ANN-MS antennas with respect to 1.56 m reference height), even with very short SNR series (about 90 min).

In fully operative scenarios, more than one low-cost instrument could be installed. Moreover, multi-constellation receivers and antennas could be selected. With large SNR observation set at hand, the definition of specific criteria for selecting and combining LSPs best suited for antenna height retrieval could help to reduce uncertainty of antenna heights and to investigate residual low frequencies in the LSP to asses the quality of the direct SNR removal step [39,40,41,42]. Moreover, suitable statistics tools could be exploited in order to increase precision of combined antenna heights. For example, the effectiveness of a weighted average of LSP peak position involving the ratio between LSP peak amplitude and LSP spread could be investigated. Areas of the Fresnel ellipses could also be considered as weighting factors.

The adoption of low-cost instruments opens the opportunity to install more instruments in a multi-point observation setup for spatially extensive GNSS-R applications. According to Equations (11), the size of a Fresnel zone increases as the antenna height increases. This fact suggests the evaluation of instrument installation on top of chairlift pylons serving glacier sky slopes for glacier monitoring in the summer. In such applications, the hypothesis of an horizontal planar reflective surface should be removed. In fact, even if it has been demonstrated [11] that this approximation leads to good results even with slope angles up to 8°, the multi-path phase difference Equation (3) can be rewritten to take into account the role of terrain slope properly described by means of a hi-resolution digital elevation model of a smooth reflective surface that would mildly change during the snow accumulation period.

## Figures and Tables

**Table 1:** Geodetic-grade instruments: differences between reference and GNSS-R based antenna heights [GNSS session March 2018].

Satellite and L-Band	Differences between Reference ($h_{0}$, $h_{1}$) and GNSS-R Antenna Heights [m]	
	Ant. AX1202GG	
	$h_{0}$ = 1.29	$h_{1}$ = 1.16
G05__L1	+0.12	
G19__L1	−0.02	
G19__L2	+0.02	
G17__L1	0.00	
G17__L2	+0.03	
G02__L1		−0.03
G02__L2		0.00
G15__L1		+0.15
G15__L2		+0.04
G06__L1		+0.02
G06__L2		+0.01
G13__L1		−0.05

**Table 2:** Low-cost instruments: differences between reference and GNSS-R based antenna heights [GNSS session February 2019].

Satellite and L-Band	Differences between Reference ($h_{0}$, $h_{1}$) and GNSS-R Antenna Heights [m]			
	Ant. TW4721		Ant. ANN-MS	
	$h_{0}$ = 1.71	$h_{1}$ = 1.56	$h_{0}$ = 1.71	$h_{1}$ = 1.56
G13__L1	+0.02		+0.08	
G06__L1	+0.02			
G23__L1	+0.15		+0.11	
G09__L1	+0.27			
G17__L1		+0.04		+0.12
G24__L1				−0.03
G12__L1		−0.18		−0.16
G19__L1		−0.07		−0.08
G05__L1		0.00		+0.03
G30__L1		+0.07		+0.19

**Table 3:** Low-cost instruments (antenna face-down): differences between reference and GNSS-R based antenna heights [GNSS session February 2019].

Satellite and L-Band	Differences between Reference ($h_{0}$, $h_{1}$) and GNSS-R Antenna Heights [m]	
	Ant. TW4721 face-down	
	$h_{0}$ = 1.68	$h_{1}$ = 1.53
G13__L1	−0.21	
G06__L1	+0.17	
G12__L1		+0.24
G30__L1		+0.21

## References

[1] Teunissen P.J., Montenbruck O. (2017). Springer Handbook of Global Navigation Satellite Systems.

[2] Awange J. (2018). GNSS Environmental Sensing.

[3] Martin-Neira M. (1993). A Passive Reflectometry and Interferometry System (PARIS): Application to ocean altimetry. ESA J..

[4] Anderson K.D. (2000). Determination of water level and tides using interferometric observations of GPS signals. J. Atmos. Ocean. Technol..

[5] Lofgren J.S., Haas R., Scherneck H.G. (2014). Sea level time series and ocean tide analysis from multipath signals at five GPS sites in different parts of the world. J. Geodyn..

[6] Roussel N., Ramillien G., Frappart F., Darrozes J., Gay A., Biancale R., Striebig N., Hanquiez V., Bertin X., Allain D. (2015). Sea level monitoring and sea state estimate using a single geodetic receiver. Remote Sens. Environ..

[7] Masters D., Zavorotny V., Katzberg S., Emery W. GPS signal scattering from land for moisture content determination. Proceedings of the IGARSS 2000—IEEE 2000 International Geoscience and Remote Sensing Symposium. Taking the Pulse of the Planet: The Role of Remote Sensing in Managing the Environment (Proceedings (Cat. No.00CH37120)).

[8] Larson K.M., Small E.E., Gutmann E., Bilich A., Axelrad P., Braun J. (2007). Using GPS multipath to measure soil moisture fluctuations: Initial results. GPS Solut..

[9] Tiuri M., Sihvola A., Nyfors E., Hallikaiken M. (1984). The complex dielectric constant of snow at microwave frequencies. IEEE J. Ocean. Eng..

[10] Larson K.M., Gutmann E.D., Zavorotny V.U., Braun J.J., Williams M.W., Nievinski F.G. (2009). Can we measure snow depth with GPS receivers?. Geophys. Res. Lett..

[11] Larson K.M., Nievinski F.G. (2012). GPS snow sensing: Results from the earthscope plate boundary observatory. GPS Solut..

[12] Koch F., Prasch M., Schmid L., Schweizer J., Mauser W. (2014). Measuring snow liquid water content with low-cost GPS receivers. Sensors.

[13] McCreight J.L., Small E.E., Larson K.M. (2014). Snow depth, density, and SWE estimates derived from GPS reflection data: Validation in the western U.S. Water Res. Res..

[14] Nievinski F.G., Larson K.M. (2014). Inverse modeling of GPS multipath for snow depth estimation, Part I: Formulation and simulations. IEEE Trans. Geosci. Remote Sens..

[15] Siegfried M.R., Medley B., Larson K.M., Fricker H.A., Tulaczyk S. (2017). Snow accumulation variability on a West Antarctic ice stream observed with GPS reflectometry, 2007–2017. Geophys. Res. Lett..

[16] Henkel P., Koch F., Appel F., Bach H., Prasch M., Schmid L., Schweizer J., Mauser W. (2018). Snow water equivalent of dry snow derived From GNSS carrier phases. EEE Trans. Geosci. Remote Sens..

[17] Li Y., Chang X., Yu K., Wang S., Li J. (2019). Estimation of snow depth using pseudorange and carrier phase observations of GNSS single-frequency signal. GPS Solut..

[18] Zhou W., Liu L., Huang L., Yao Y., Chen J., Li S. (2019). A new GPS SNR-based combination approach for land surface snow depth monitoring. Sci. Rep..

[19] Egido A., Caparrini M., Ruffini G., Paloscia S., Santi E., Guerriero L., Pierdicca N., Floury N. (2012). Global navigation satellite systems reflectometry as a remote sensing tool for agriculture. Remote Sens..

[20] Beckmann P., Spizzichino A. (1987). The Scattering of Electromagnetic Waves From Rough Surfaces.

[21] Baghdadi N., Zribi M. (2016). Microwave Remote Sensing of Land Surface.

[22] Jin S., Cardellach E., Xie F. (2014). GNSS Remote Sensing.

[23] Georgiadou P., Kleusberg A. (1988). On carrier signal multipath effects in relative GPS positioning. Map Collect..

[24] Ge L., Han S., Rizos C. (2000). Multipath mitigation of continuous GPS measurements using an adaptive filter. GPS Solut..

[25] Ray J.K., Cannon M.E. (2001). Synergy between global positioning system code, carrier, and signal-to-noise ratio multipath errors. J. Guid. Control Dyn..

[26] Bilich A., Larson K.M. (2007). Mapping the GPS multipath environment using the signal-to-noise ratio (SNR). Radio Sci..

[27] Zhong P., Ding X., Yuan L., Xu Y., Kwok K., Chen Y. (2009). Sidereal filtering based on single differences for mitigating GPS multipath effects on short baselines. J. Geod..

[28] Kos T., Markezic I., Pokrajcic J., Grgic M., Bozek J., Grgic S. (2010). Effects of multipath reception on GPS positioning performance. Proceedings of the ELMAR-2010.

[29] Zimmermann F., Schmitz B., Klingbeil L., Kuhlmann H. (2018). GPS multipath analysis using fresnel zones. Sensors.

[30] Estey L., Wier S. (2014). Teqc Tutorial: Basics of Teqc Use and Teqc Products.

[31] Estey L., Wier S. (2016). GFZRNX 1.05 User Guide.

[32] Lomb N.R. (1976). Least-squares frequency analysis of unequally spaced data. Astrophys. Space Sci..

[33] Scargle J.D. (1982). Studies in astronomical time series analysis. II—Statistical aspects of spectral analysis of unevenly spaced data. Astrophys. J..

[34] Press W.H., Rybicki G.B. (1989). Fast algorithm for spectral analysis of unevenly sampled data. Astrophys. J..

[35] Shumway R.H., Stoffer D.S. (2017). Time Series Analysis and Its Applications.

[36] VanderPlas J.T. (2018). Understanding the lomb-scargle periodogram. Astrophys. J. Suppl. Ser..

[37] Roesler C., Larson K.M. (2018). Software tools for GNSS interferometric reflectometry (GNSS-IR). GPS Solut..

[38] Hristov H. (2000). Fresnel Zones in Wireless Links, Zone Plate Lenses and Antennas.

[39] Yu K., Ban W., Zhang X., Yu X. (2015). Snow depth estimation based on multipath phase combination of GPS triple-frequency signals. IEEE Trans. Geosci. Remote Sens..

[40] Tabibi S., Geremia-Nievinski F., van Dam T. (2017). Statistical comparison and combination of GPS, GLONASS, and Multi-GNSS multipath reflectometry applied to snow depth retrieval. IEEE Trans. Geosci. Remote Sens..

[41] Wang X., Zhang Q., Zhang S. (2017). Water levels measured with SNR using wavelet decomposition and Lomb-Scargle periodogram. GPS Solut..

[42] Chen F., Liu L., Guo F. (2019). Sea surface height estimation with Multi-GNSS and wavelet de-noising. Sci. Rep..

